# Dynamics of Water Intrusion in Polyelectrolyte-Bound Kaolinite: An Insight into Durability Mechanisms via Atomistic Modeling

**DOI:** 10.1021/acsomega.3c10385

**Published:** 2024-05-22

**Authors:** Javier A. Grajales, Dallas N. Little, John F. Rushing

**Affiliations:** † College of Engineering, Zachry Department of Civil and Environmental Engineering, Texas A&M University, 3136 TAMU, College Station, Texas 77843-3136, United States; ‡ Centro Experimental de Ingeniería, Universidad Tecnológica de Panamá, Ciudad de Panamá 0819-07289, Panamá; § U.S. Army Engineer Research and Development Center, Vicksburg, Mississippi 39180, United States

## Abstract

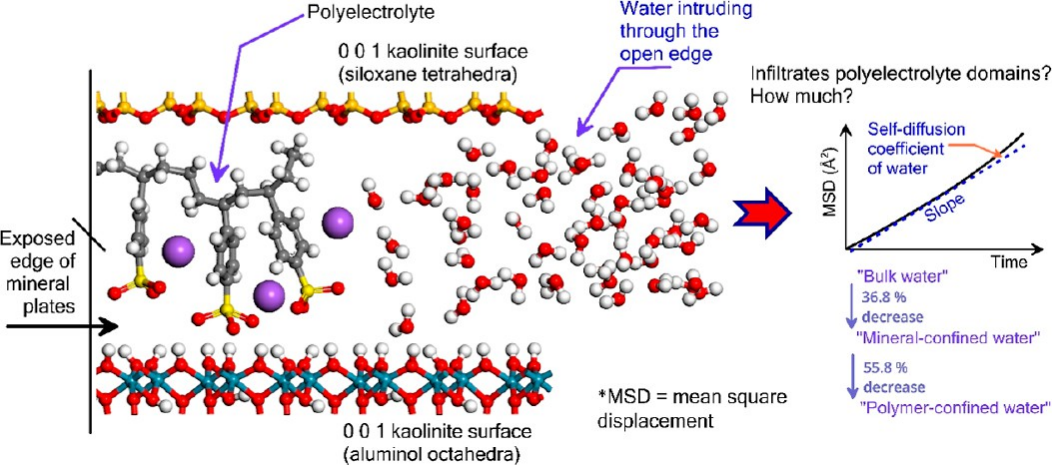

This research addresses interaction mechanisms of water-soluble polymers used as soil mineral stabilizers via atomistic classical molecular dynamics (MD). Specifically, this study addresses polyelectrolyte interactions with kaolinite, a ubiquitous clay mineral, in soils. The two water-soluble polymeric species evaluated are PSS: poly(4-sodium styrenesulfonate) and PDADMAC: poly(diallyldimethylammonium chloride). The primary focus is the evaluation of water migration through a polymer-kaolinite composite system, the resulting molecular arrangement and interactions, and the extents of water migration through the polymeric phase-binding kaolinite interfacial planes. Mean square displacement (MSD) analysis was used to quantify the motion of the system species from the MD trajectories by calculation of self-diffusion coefficients and comparison of the curves obtained. The MD results indicate that water infiltrates the polyelectrolyte phase adhering to the mineral interfaces. Nevertheless, the MSD analysis results indicate a 55.8% reduction in water self-diffusion with respect to pure mineral-confined water. This is a compelling indication that polyelectrolytes can hinder water movement. Most importantly, MSD analysis of both polyelectrolyte species shows that the movement of the chains is negligible relative to that of water. These results strongly suggest that the movement of polymer phases is restricted only to local chain mobility and a rather bound state to the mineral surfaces prevails.

## Introduction

Organic polymers are emerging alternatives as chemical stabilizers for enhancing soil-based composites^1−6^ when compared to traditional stabilizing compounds of a cementitious/pozzolanic nature such as Portland cement and hydrated lime.^7−12^ As a historical reference, Moore^13^ provides evidence of the use of lime/pozzolan mixes dating to the ages of the Roman Empire. The most significant advantage of traditional stabilizing compounds is the long-term hardening achieved through the formation of hydration products that develop an increasing strength over time within the composite interstices and a level of resistance to environmental factors such as exposure to water due to the dense nature of the stabilized material product. However, a major disadvantage is that these materials are brittle.^14^ Significant advantages of polymer-soil composites include mitigation of the erodibility of loose soil particles,^2,15^ improved compressive strength, fracture toughness, and resistance to fatigue as opposed to the brittle nature of cementitious composites.^14,16,17^ Furthermore, all these characteristics of polymers develop at low dosages (2 wt %) versus 4 wt % and above, which are typical dosages for traditional stabilizers. However, many polymer species that have been attempted as stabilizers in soils are water-soluble. The hydrophilic characteristics of water-soluble polymers can be advantageous in multiple ways, but they also make them vulnerable to water incursions after the fabrication of the polymer-soil composite. The advantages are, first, the ease of mixing because water acts as the “carrier” of the polymer chains while simultaneously enhancing the packing ability of soil mineral particles. Second, the curing mechanism for polymer-stabilized composites is simple; it only involves the drying of the interstitial water “breaking away” for the functional groups present in the polymer chains to bind directly to the soil mineral surfaces exposed.^14,16,17^ Third, the curing time required for polymer-soil composites is not dependent on the kinetics of reactions between stabilizer and native soil minerals, as is needed for most cementitious/pozzolanic stabilizers. Last, because of the commonly nonreactive binding nature (adhesive binding) of many polymer species used, polymers may be less restricted by the availability of soil minerals as raw reactants dictated by the geochemistry of the environment.

While the aforementioned resistance to erodibility of the polymer phases would not compromise the polymer-soil composite due to the strong adsorption that is often achieved with the mineral substrate,^18^ the durability of the composite “as a whole” could be transiently compromised by the infiltration of water wetting the interparticle interstices and composite interfaces. This is the major potential disadvantage of polymer-soil composites. However, a quantifiable mechanism of what occurs inside the polymer chain matrix of water-soluble polymer-soil composites in a wetting event has not been defined. The reason for this gap in knowledge is that polymers are long macromolecular species that exhibit complex, nonreactive conformation-related behaviors that do not exist in the case of inorganic salts or crystals. Polymers pose a structure-behavior entropic dependency as well as a multiplicity of contacts with surfaces, which causes an amplification of molecular binding. A consequence of such strong binding is that once adsorbed, a polymer chain cannot easily desorb from the surface due to the prohibitively high energetic barrier for desorption, which requires simultaneous detachment of all monomeric units.^18^ This atomistic modeling study seeks to provide insights into these phenomena when water is introduced into the system.

As previously defined, two polyelectrolytes were chosen as representative model species of water-soluble polymers: poly(4-sodium styrenesulfonate), or PSS, as a representative model species with anionic charged sites and poly(diallyldimethylammonium chloride), or PDADMAC, as a model with cationic charged sites. Kaolinite is chosen as a representative mineral as it is ubiquitous in clayey^19,20^ nonexpansive and weak soils.^21^ The core contribution this atomistic modeling study seeks to disseminate is the explicit definition and quantification of diffusion mechanisms at the polymer-mineral binding domain resulting from the interfacial intermolecular interactions among polymer, mineral, and water system species. To do so, using classical molecular dynamics (MD), this study describes the phenomenon in a qualitative (visual) fashion, obtained from a mechanistic simulation approach. In a bifold way, MD-computed molecular motion data are used quantitatively via mean square displacement (MSD) dynamic analysis to provide self-diffusion coefficients and the characteristics of self-diffusion behavior from MSD curves. Results for the self-diffusion of water are compared with values from the TIP3P^22^ explicit water model.

## Modeled System and Component Species

**Figure 1:**
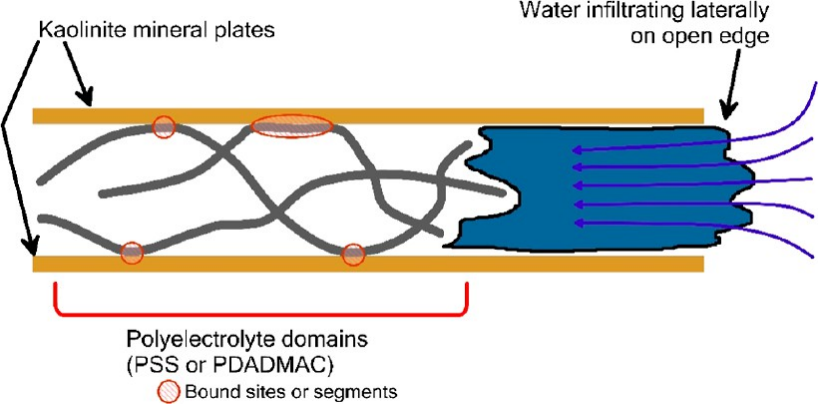
Schematic of the water infiltration process at the open edges of the kaolinite plates.

The schematic shown in Figure 1 depicts the idea modeled consisting of surfaces of kaolinite plates that are bound by a polymeric matrix on their basal plane (0 0 1), leaving open edges open to water intrusion.

**Figure 2:**
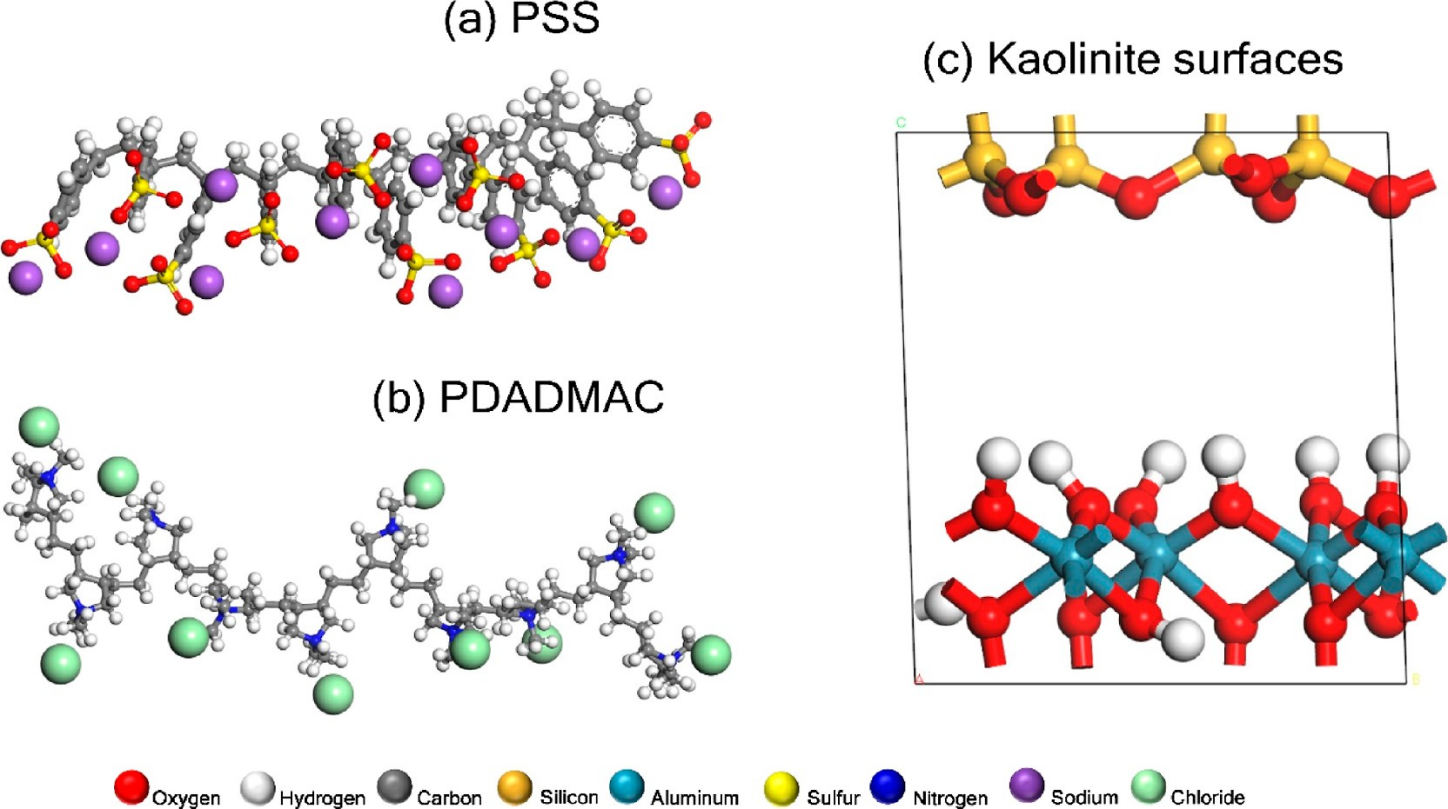
Structure and composition of modeled molecular and crystalline species: (a) PSS, (b) PDADMAC, and (c) kaolinite mineral surfaces.

Figure 2 illustrates the model molecular structure of the polyelectrolyte species: (a) PSS and (b) PDADMAC. Figure 2c illustrates the modeled structure of the kaolinite soil mineral.

## Research Methodology

### Theoretical Constructs

Classical molecular mechanics^23^ is the general theoretical basis for this study, encompassing both static and dynamic modeling methods. Static methods include single-point energy calculations and structure relaxation. Dynamic simulation consists of MD, and the analysis and calculation of diffusive behavior and coefficients are obtained via MSD dynamic analysis. A brief theoretical introduction to MD and MSD techniques is given next, respectively.

#### MD Simulations

The movement of atoms and molecules in a physical system is calculated over a time span of interest by solving Newton’s second law of motion as the governing equation (eq 1), where for each atom in the system, *F* is the force, *r* is the position, *V* is the potential energy, and *t* is the time. The second-order differential equation is solved iteratively for each atom in the system based on a series of interatomic energy potentials that account for the different modes of motion each atom can possibly have. Such a series of potentials is denominated a force field, forming an expression that is the kernel of any classical molecular modeling approach. As an exemplification, a basic force field expression is formulated in (eq 2), and every term in the expression is formally denominated a functional form.
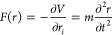
1
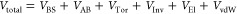
2

Where *V*_total_ is the total energy of the system, *V*_BS_ corresponds to bond stretch, *V*_AB_ represents the angle bend, *V*_Tor_ denotes torsion dihedrals, *V*_Inv_ corresponds to out of plane motions, *V*_El_ denotes electrostatic interactions, and *V*_vdW_ accounts for van Der Waals force components. It is important to remark that *V*_El_ and *V*_vsW_ components of energy can account for the motion, attraction, or repulsion among both bonded (intramolecular) and nonbonded (intermolecular) atom interactions.

#### MSD Dynamic Analysis

This technique is implemented to calculate the self-diffusion coefficient^24^ of chemical species composing materials at the molecular level and quantify their averaged movement. The physical basis for the MSD analysis is Einstein’s equation (eq 3).
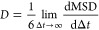
3

Einstein’s equation^25^ approaches the quantification of the atomic motion over a time span as the limit of the derivative of the MSD (in Å^2^) with respect to a discrete time step (Δ*t*) when the time step tends to infinity (Δ*t* → ∞). It also accounts for the 6 degrees of freedom that can occur in atomic motion spatially (1/6 quotient in the equation). Consequently, based on Einstein’s equation, the MSD analysis can use as input the calculated motions of atoms in molecules in every time step of a MD trajectory. The importance of this is that the entire approach can quantify the motion of matter, including an explicit representation of chemical species in a physical system with defined atomic compositions, structures, conformations, and molecular size.

### Computational Overview

This study implemented the interface force field (IFF) for all of the calculations. The reason why the IFF^26,27^ is used is that the molecular system includes both organic (polyelectrolyte) and inorganic (kaolinite mineral) types of chemical species. Many force fields only have the capability of handling one type of chemical species but not both. Examples of these are the universal force field (UFF)^28^ and the polymer consistent force field (PCFF).^29^ The IFF is designed as a flexible platform that merges parameters from some force fields for organics with parameters for inorganic species. For example, this study uses the IFF version that combines the parameters from PCFF for organics with the parameters for inorganics provided by IFF. All calculations were conducted using 3DS Biovia Materials Studio 2022 (MS2022) as a force field driver and supercell construction platform.

### Construction of Molecular and Crystalline Systems

Polymeric phases were constructed with the Amorphous Cell module available in MS2022. As a first step, amorphous supercells containing the polyelectrolytes dissociated in aqueous solution were constructed. For purposes of simulating a typical concentration value common in commercial solutions, concentrations of built supercell models were set to approximate 20 wt % dilution in water. A negligible concentration difference exists between PSS and PDADMAC aqueous supercells due to their different unit molecular weights. However, this does not cause a significant impact on the results as the systems are initially dried in full and exposed to the same amount of water for the evaluation of rewetting. For the case of PSS, a system was constructed in a cubic supercell emulating the weight ratio with equivalent molecular counts of 10 PSS chains (10 units long), 100 sodium ions (Na^+^), and 2700 water molecules. A similar system was constructed for PDADMAC containing 10 chains (10 units long), 100 chloride anions (Cl^–^), and 2700 water molecules. While the packing is amorphous in these supercells, they are periodic in all directions. In the case of the kaolinite mineral crystal lattice, the construction of a slab model from a unit cell was not necessary. Instead, a kaolinite slab model cleaved along the basal plane (001) was obtained from the IFF’s mineral structure database, and its dimensions were modified to accommodate the polymeric phases as well as the volume occupied by water molecules intruding laterally.

### MD Simulation Workflow

#### Preliminary Structure Relaxation

Structure relaxation is a necessary first step to ensure that the model species are at their potential energy minima. At their minima, the position of the atoms will change to yield the bond lengths and angles that correspond to the minimum energy state in the potential energy surface (PES) of the molecule.^23^ The entire conformation and geometry of the molecule change in this process. Performing a structure relaxation on all the species to be modeled is necessary to prevent surpassing the initial energy deviation threshold when conducting MD runs. Such an energy deviation threshold is the upper allowable limit that the MD algorithm permits on the initial trajectory step as the equilibration of temperature and initial random velocities initiates. Structure relaxation was conducted on each molecular constituent modeled, starting from the polyelectrolyte monomers and chains, the construction of aqueous polymer supercells, the kaolinite mineral lattice, and the interfacial polymer-mineral supercell system. The minimization algorithm used for the structure relaxation conducted in this study is denominated Smart. This specific algorithm is available as part of the Materials Studio Forcite module. The Smart minimizer is robust and comprehensive because it is a cascade type. That is, it has the capability to use other algorithms (steepest descent, ABNR, and quasi-Newton), all combined for a more agile minimization and more reliable energy minima in the structure relaxation and geometry optimization process.

#### MD Setup

Basic MD parameters used in the simulations include a time step of 1 fs and the use of the velocity Verlet integration algorithm. Energy parameters include the use of Ewald summation for electrostatics with an accuracy of 1 × 10^–5^ kcal/mol, a cutoff of 12 Å, and a buffer width of 0.5 Å. The van der Waals energy component was accounted for as atom-based with a cubic spline method, a cutoff distance of 18.5 Å with long-range correction, and a buffer width of 2 Å. The values used for functions such as temperature and pressure depend on the thermodynamic ensemble used. At different stages in this study, two ensembles were used: *NPT* (constant number of molecules, constant pressure, and constant temperature) and *NVT* (constant number of molecules, constant volume, and constant temperature). For both ensembles used, the temperature was regulated with a thermostat implementing the Nose method with a *Q* ratio of 0.01. In the case of the *NPT* ensemble, the Parrinello method was implemented to incorporate a barostat set to maintain a pressure of 1 atm on the cell walls with a cell time constant of 1 ps, while all cell dimensions and angles varied to accommodate the change in volume. When the *NVT* ensemble is used, the only constraint imposed on the system is that of its inherent constant volume. The *NVT* ensemble was used only on systems that had gone through a prior *NPT* run in which the volume was already stabilized. In this way, the interactions of the system constituents and their diffusive behavior were examined, excluding any volumetric variation effect.

#### Emulation of Initial Dry-Curing Conditions via MD

**Figure 3:**
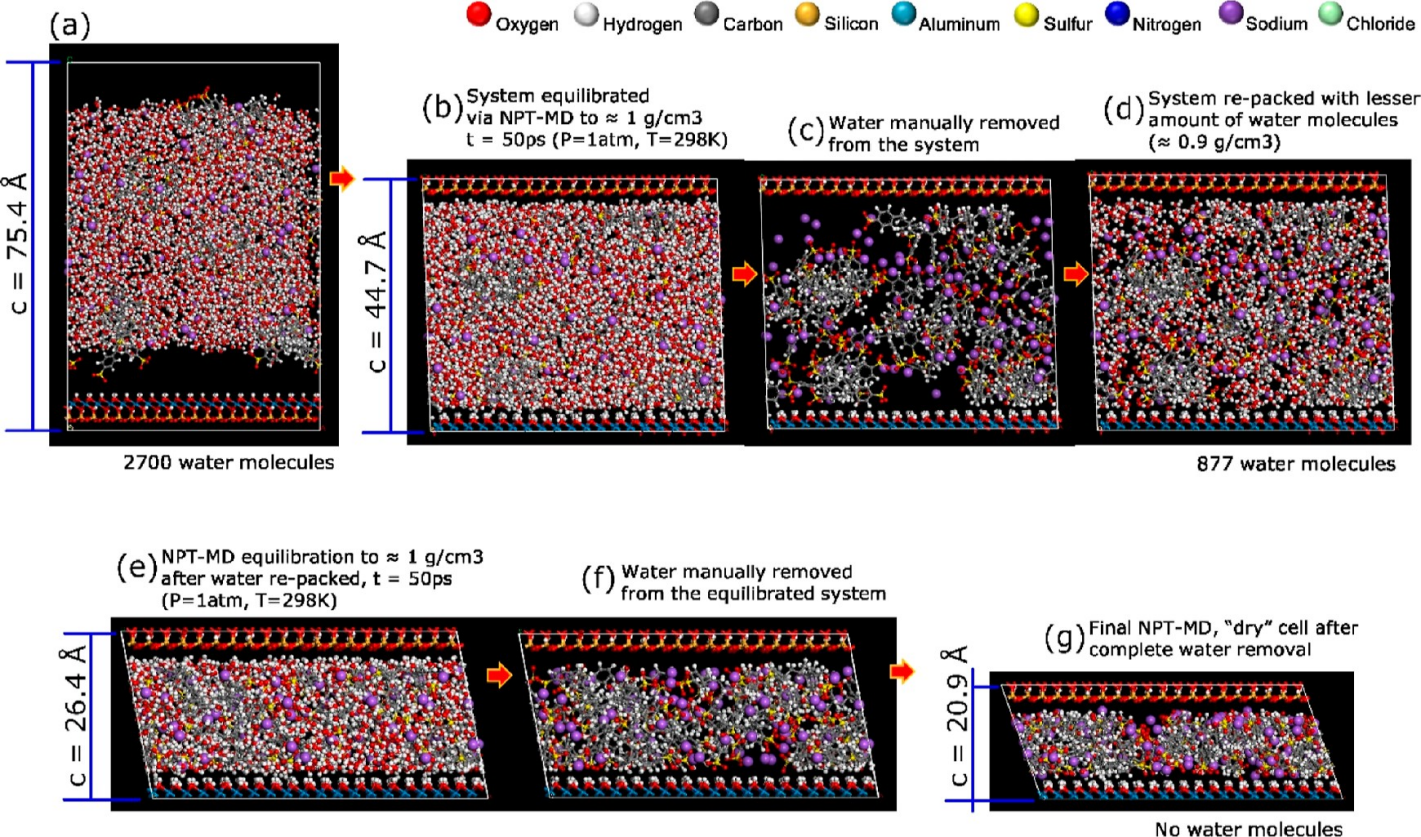
MD trajectory frames were used to simulate the dry binding mechanism of PSS to the kaolinite interfaces upon water depletion.

**Figure 4:**
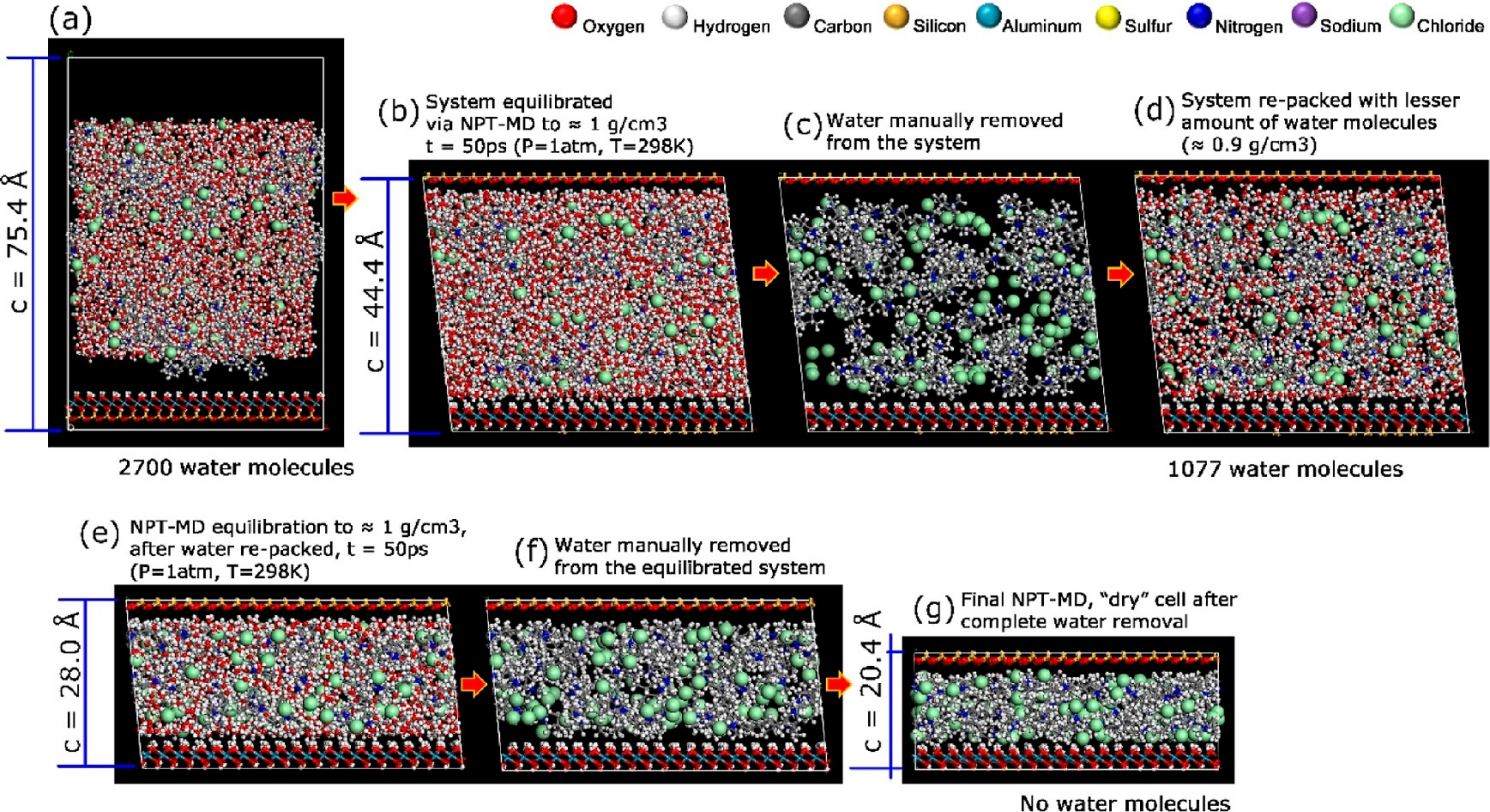
MD trajectory frames are used to simulate the dry binding mechanism of PDADMAC to the kaolinite interfaces upon water depletion.

As explained previously, the curing of polymer-soil composites consists of drying the material to allow the aqueous phase to “break away” due to evaporation induced by dry air conditions and/or heat. In this study, this effect is emulated by conducting a series of MD simulations implementing the *NPT* ensemble in a three-stage *NPT* sequence equally conducted for both polyelectrolyte species. Taking PSS as an example, a first step is an initial equilibration of the kaolinite supercell system with 20% wt. aqueous polyelectrolyte solution, shown in Figure 3a,b. The second stage is a subsequent “manual” removal of water molecules from the supercell and repacking with a decreased density (0.9 g/cm^3^), followed by re-equilibration with a second NPT-MD (Figure 3c–e). The third and final stage is then the removal of all water molecules and *NPT*-MD equilibration to obtain a dry-condition polyelectrolyte-mineral composite supercell (Figure 3f–g). The described modeling sequence was implemented for PDADMAC as well (Figure 4a–g).

#### Dynamic Simulation of Water Ingress via MD

**Figure 5:**
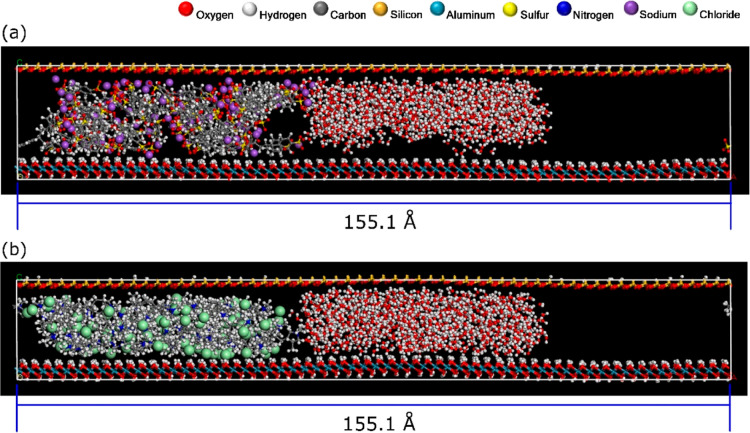
Interfacial composite system with kaolinite surfaces extended laterally to accommodate a water body incoming from the edge gap (cross-sectional view).

Dry-condition equilibrated supercells (Figures 3 and 4g) were set as the initial physical systems to characterize an aqueous ingress event. To do this, the supercells were modified by extending the lateral edge in one direction, creating a long, hollow space large enough to accommodate a body of water along with a void gap. This gap was set with a sufficiently large volume to isolate the water body from mirroring periodic replicates and allow unidirectional flow through the polyelectrolyte matrix (Figure 5a,b). The lateral incursion of water was modeled for a time frame of 10 ns.

The MD ensemble chosen to model the ingress of water was *NVT*. The choice of the *NVT* ensemble was necessary to prevent the supercell from changing dimensions by restraining the movement of the kaolinite mineral plates vertically (orthogonal to the basal planes). Thus, this allows simulation of the lateral movement of the aqueous phase as it pushes through the adsorbed polyelectrolytes. A total of 948 water molecules were packed to ingress into the polyelectrolyte systems. To start the simulation with equal conditions for both polymeric species, the same water body was packed on one system and used for both polymeric species. Both water-packed systems were structurally relaxed before the MD simulations.

### Methodology for Calculations of Self-Diffusion Coefficients

#### MSD Analysis

MD trajectories were analyzed to determine the self-diffusion coefficients of the different chemical species in the system. In this case, the species of interest are water and the polyelectrolyte matrix, for which self-diffusion coefficients (DH_2_O for water) and polyelectrolyte chains (DP) were calculated. The importance of the latter is to provide metrics of the self-diffusive behavior of the polymeric phases as water ingresses and interacts with both mobile and mineral-bound polyelectrolyte domains over the time frame of the simulation.

### Baselines for Water Dynamics

**Figure 6:**
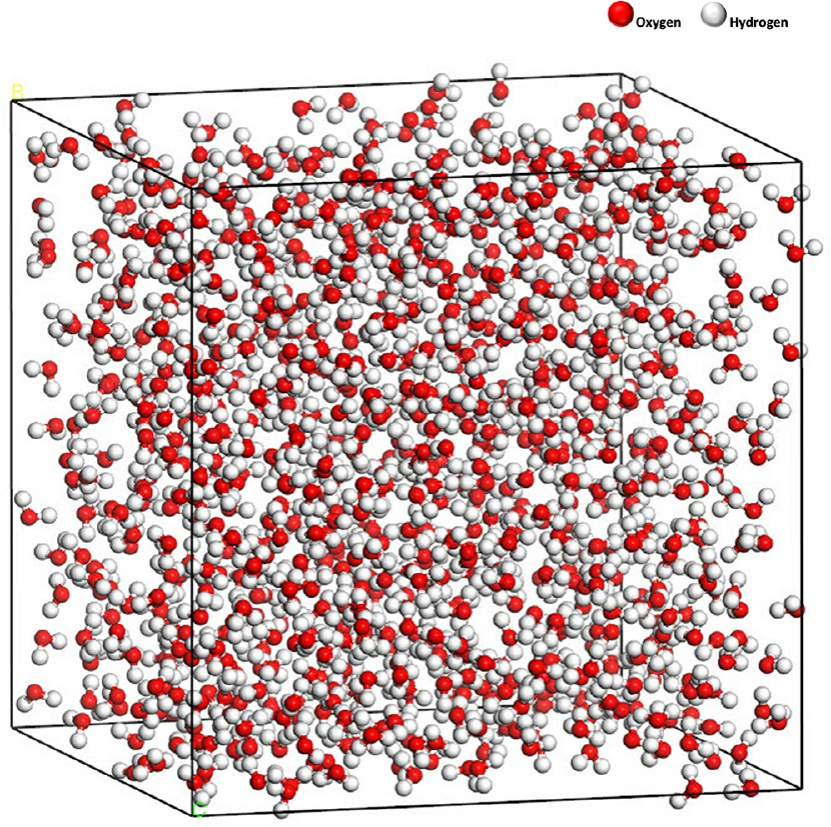
Cubic box containing 948 water molecules.

**Figure 7:**
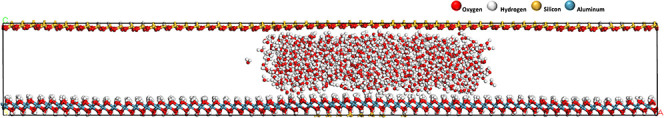
Kaolinite-confined water system containing 948 water molecules.

In order to systematically determine the effects of confinement within mineral plates on water diffusion, a cubic box was constructed containing 948 water molecules to calculate the self-diffusion of bulk water molecules (Figure 6). The box was initially relaxed via a geometry optimization. Subsequently, a short (50 ps) *NPT* dynamics using the Berendsen barostat was conducted to reach a pressure equilibrium of 1 atm while still maintaining the cubic shape. Following this, a 1 ns *NVT* run was conducted, from which the self-diffusion coefficient of bulk water was obtained. The self-diffusion of kaolinite-confined pure water was calculated via a 1 ns *NVT* run on which only the water and mineral phases were part of the system (Figure 7).

## Results and Discussion

### Overview of MD Trajectories

**Figure 8:**
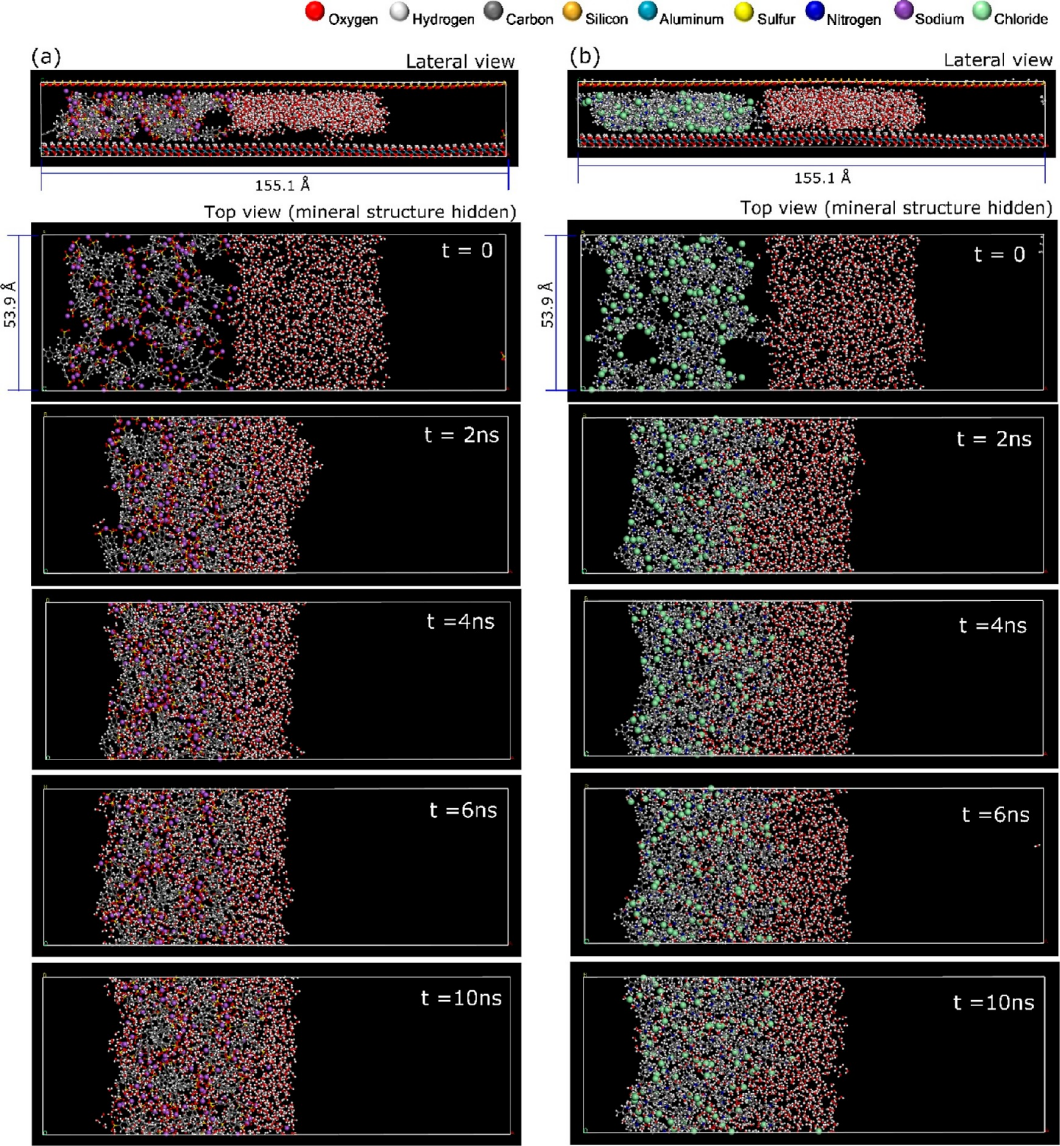
Lateral and top view snapshots of MD trajectories for (a) PSS and (b) PDADMAC (mineral structure is hidden to facilitate visual tracking of the polyelectrolyte and water species).

A general observation of the MD trajectories indicates that water can fully infiltrate and saturate the polymeric chain matrix for both polyelectrolyte species that have been modeled. Nonetheless, two other important behaviors within the 10 ns simulation time frame were observed. First, for the most part, water molecules that infiltrate the polymeric chain matrix do not flow out: once inside, water molecules still self-diffuse but with significantly restricted molecular and translational motion hindered by the matrix of polymeric chains. Second, not all of the mass of water molecules modeled was able to penetrate inside the polymeric chain matrix. These two observations can be visually verified in Figure 8a,b, which shows the progression of the MD trajectories as a series of snapshots over time.

### Diffusivity of Water

**Figure 9:**
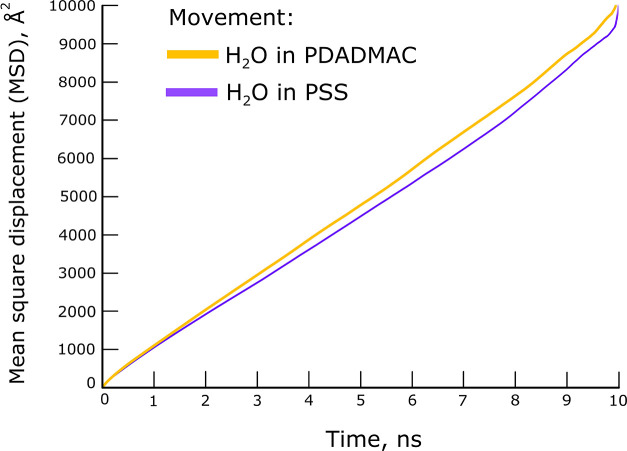
MSD curves of water for polyelectrolyte species binding to kaolinite surfaces.

The MSD curves for water molecules are shown in Figure 9. The curves are linear throughout the entire simulation time, indicating the ability of water molecules to freely diffuse within the polymeric chain matrix. The self-diffusion coefficient of water (DH_2_O) for each polyelectrolyte system is presented in Table 1.

**Table 1:** Modeled Self-Diffusion Coefficients of Water in Polyelectrolyte Systems Studied and Comparison with Modeling Values Available in the Literature

chemical species	self-diffusion coefficient of water (DH_2_O), cm^2^/s	
	this study	TIP3P model^22^
pure water (bulk)	5.98 × 10^–5^	5.8 × 10^–5^
pure water (mineral-confined)	3.78 × 10^–5^	
poly(4-sodium styrenesulfonate)	1.67 × 10^–5^	
poly(diallyldimethylammonium chloride)	1.69 × 10^–5^	

Table 1 shows that the water self-diffusion coefficient values obtained are nearly the same for the polyelectrolyte species investigated. This numerically corroborates the water susceptibility potential of both polyelectrolyte–kaolinite systems. In addition, Table 1 lists the baseline self-diffusion calculations of bulk water and mineral-confined pure water. The most important finding is that the bulk water self-diffusion coefficient calculated in this study closely approaches that of the TIP3P^22^ model, which sets a solid validation reference point for relative comparisons and trends. Furthermore, reduction percentages were calculated. Results for these indicate a 36.8% reduction of water bulk self-diffusion upon mineral confinement and a 55.8% reduction of water self-diffusion solely due to the presence of the polyelectrolytes in the system with respect to the mineral-confined former.

### Diffusivity of Polyelectrolytes

The self-diffusion coefficient of water (DH_2_O) as it penetrates both polyelectrolyte matrices (PSS and PDADMAC) has been addressed. However, as water diffuses into these, a simultaneous process occurs with the polymer chains: the self-diffusion of the chains as their structural conformations accommodate the water intrusion.

The self-diffusion coefficient values for the polyelectrolyte chain species are presented in Table 2. The value obtained for PSS is on the order of 10^–7^ cm^2^/s. For PDADMAC, the value is 1 order of magnitude smaller, 10^–8^ cm^2^/s. The reason for this difference likely arises from the conformations inherent in their composition. For example, styrene rings in PSS are planar, which increases the space for self-diffusive motion. In the case of PDADMAC, a five-membered cyclic structure conforms the backbone more rigidly bonded, hindering its self-diffusion more prominently. Experimental precedents addressing the self-diffusion of polymers in a confined system such as the kaolinite 0 0 1 interfaces have not been found for comparison. However, the dominant relative trend is that the degree of movement of the polyelectrolyte species has an impact on the movement of water as there is an additional energy exertion demanded by the conformational changes of the chains, retarding the “free” undisturbed movement of water molecules along the kaolinite mineral plate gap.

**Table 2:** Modeled Self-Diffusion Coefficients of Polyelectrolyte Systems

polyelectrolyte species	self-diffusion coefficient *D*_P_, cm^2^/s
poly(4-sodium styrenesulfonate)	1.50 × 10^–7^
poly(diallyldimethylammonium chloride)	1.95 × 10^–8^

**Figure 10:**
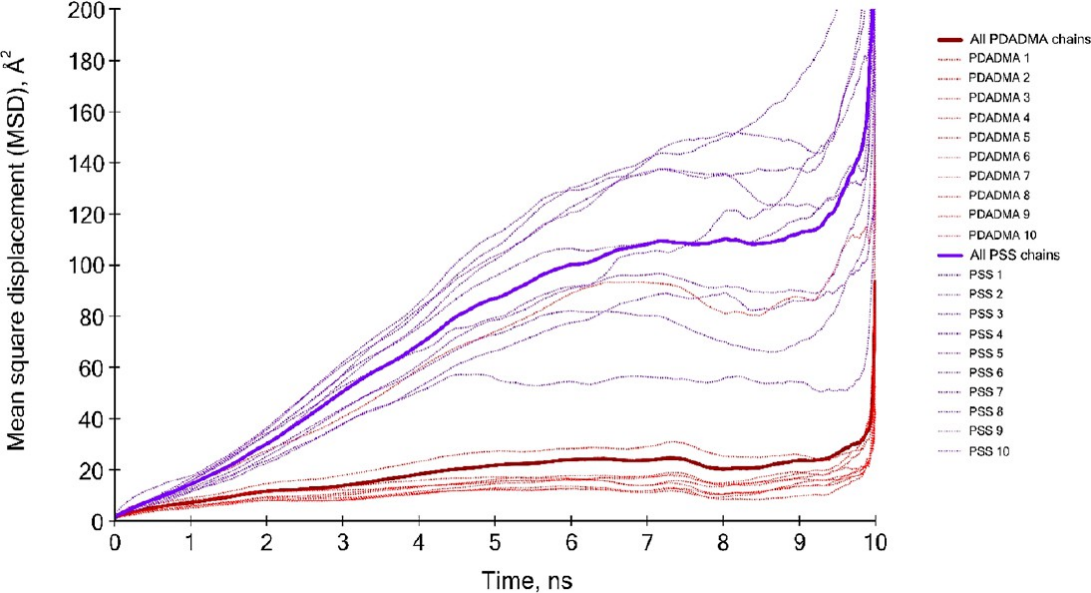
Comparison of single-chain MSD curves with averaged curves for both polyelectrolyte species.

To better understand the difference found in the chain self-diffusion coefficient of the polyelectrolyte species evaluated, the MSD curve of every single chain in the polyelectrolyte systems was evaluated and compared to the average curves (Figure 10).

When the average curves are observed, it is evident that the magnitude of the MSD values for PSS chains is larger relative to PDADMAC. Thus, PSS chains are much more mobile. In addition, when the individual-chain range of movement for PSS is examined, considerable scattering occurs. Conversely, the MSD values of PDADMAC curves are rather smaller in magnitude (overall less mobility), and their individual-chain MSD curve scattering is much less prominent.

### Weighing the Diffusivity of All System Species

**Figure 11:**
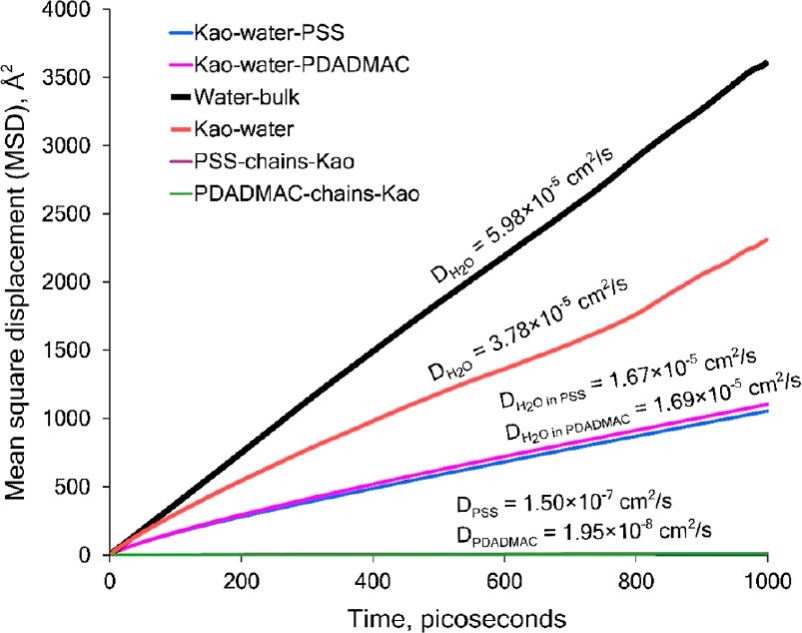
Comparison of MSD curves and self-diffusion coefficients for all species studied over a time span of 1000 ps (1 ns).

**Figure 12:**
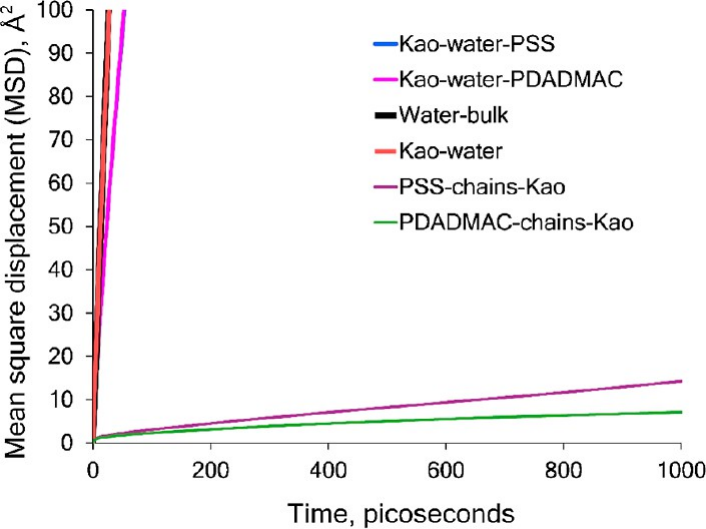
Comparison of the differences in the MSD curves of all species.

The self-diffusion coefficient of water (DH_2_O) as it penetrates both polyelectrolyte matrices (PSS or PDADMAC) has been addressed, along with a detailed characterization of the self-diffusion of these polyelectrolytes. Nevertheless, a consolidated comparison is needed to establish a systematic point of reference. For this reason, MSD results corresponding to a 1 ns MD trajectory were gathered from all system species to weigh their behavior in a normalized scheme (Figure 11). Upon observation of the chart in Figure 11, it is possible to corroborate that the movement of the polymer species is in fact negligible with respect to that of water in the different conditions evaluated (bulk, mineral-confined, and mineral-polymer-confined), which corroborates the restricted movement of the chains and their bound state to the mineral substrate. For further evidence of the existing polyelectrolyte MSD curves, Figure 12 shows an enlarged view of the chart up to 100 Å^2^ to appreciate the level of deviation of the polyelectrolyte diffusivity from that of water.

### Quantification of the Mass of Water Diffused

**Figure 13:**
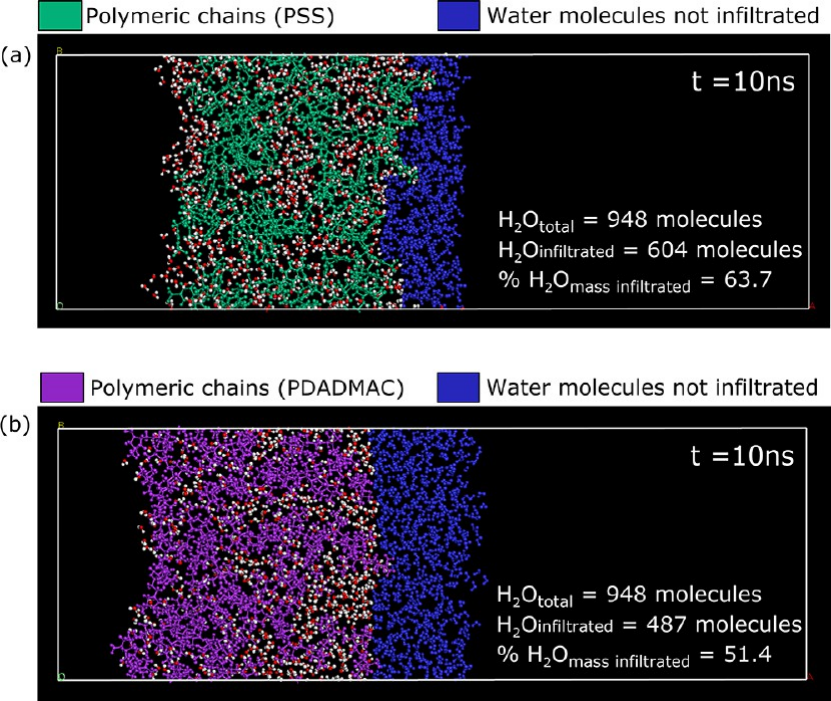
Top view of the last MD frame for each water–polyelectrolyte system. Water molecules that are not able to infiltrate the polymeric systems are colored blue.

The percentage mass of water infiltrated within the polymeric matrix was calculated via a molecule count. As previously mentioned, most of the water molecules that infiltrated the polymeric chain matrix could not “flow out” and were rather trapped inside, with some level of mobility in larger interstices. More importantly, not all the water molecules packed to ingress the polymeric systems were able to diffuse inward (this is visually conspicuous in Figure 13). Therefore, the last MD trajectory frame (10 ns) of each polyelectrolyte species was used to perform a molecule count. In the case of the PSS system, the mass of infiltrated water was 63.7%, whereas in the case of PDADMAC, it was 51.4%. These results mark a difference of 12.3%, indicating that PDADMAC is more stable in hindering the advancing front of water molecules.

When the mass of polyelectrolyte chains modeled is analyzed, PSS (unit *M*_w_ = 206.2 g/mol) is heavier than PDADMAC (unit *M*_w_ = 161.67 g/mol). This could lead to the inference that PSS would hinder more effectively the movement of water than PDADMAC given that the chain length modeled is the same for both species (10 repeating units). Conversely, the results obtained show strong indication that molecular conformation and array are dominating factors on the amount of water infiltrated. Such observation links back consistently with the differences found in self-diffusion coefficients and MSD curves of polymer chains.

## Conclusions

The general conclusion of this work is that water will infiltrate polyelectrolyte chain systems that are binding kaolinite mineral plates. However, specific important observations are:

- The self-diffusion coefficient of water in the presence of both polyelectrolyte species decreases by 55.8% when compared to that of pure kaolinite-confined water. This indicates that even though the polyelectrolytes are water-soluble species, a considerable level of hindrance to the movement of water is achieved in the bound kaolinite system.
- The self-diffusivity of polyelectrolyte chains is negligible, indicating that only very restricted movement occurs, likely due to their bound state to the mineral surface.
- Not all water molecules introduced to the systems were able to intrude the polymeric matrix within the time span of the simulation (10 ns), and a quantification of the mass of water infiltrated indicated a range of 51 to 64% infiltration of the total mass of water modeled.

In summary, although water penetrates polymeric binding domains, aqueous polymers with charged sites (polyelectrolytes) are an advantageous alternative as stabilizers of soil minerals, such as kaolinite, due to the level of hindrance to water migration found and the resistance of the polymeric chains to translational motion away from their binding interfaces. Further investigations are ongoing to extend the MD simulation time span and explore the effects of temperature on the dynamics of the system species.
